# Impact of vitamin A transport and storage on intestinal retinoid homeostasis and functions

**DOI:** 10.1016/j.jlr.2021.100046

**Published:** 2021-02-13

**Authors:** Maryam Honarbakhsh, Aaron Ericsson, Guo Zhong, Nina Isoherranen, Chengsheng Zhu, Yana Bromberg, Charlene Van Buiten, Kiana Malta, Laurie Joseph, Harini Sampath, Atreju I. Lackey, Judith Storch, Costantino Vetriani, Michael L. Chikindas, Paul Breslin, Loredana Quadro

**Affiliations:** 1 Department of Food Science, Rutgers University, New Brunswick, NJ, USA; 2 Department of Veterinary Pathobiology, University of Missouri Metagenomics Center, University of Missouri, Columbia, MO, USA; 3 Department of Pharmaceutics Health Sciences, University of Washington, Seattle, WA, USA; 4 Department of Biochemistry and Microbiology, Rutgers University, New Brunswick, NJ, USA; 5 Department of Food Science and Human Nutrition, Colorado State University, Fort Collins, CO, USA; 6 Department of Pharmacology and Toxicology, Rutgers University, Piscataway, NJ, USA; 7 Department of Nutritional Sciences, Rutgers University, New Brunswick, NJ, USA; 8 Rutgers Center for Lipid Research and Institute of Food Nutrition and Health, Rutgers University, New Brunswick, NJ, USA

**Keywords:** colon, gut microbiome, lecithin:retinol acyltransferase, retinoic acid, retinol-binding protein, vitamin A, vitamin A deficiency, IL, interleukin, LRAT, lecithin:retinol acyltransferase, *Lrat*^*−/−*^, lecithin:retinol acyltransferase–deficient mice, *Muc*, mucin, NIRF, near-infrared fluorescence, OTU, operational taxonomic unit, PAS, Periodic Acid-Schiff, PCA, principal component analysis, PERMANOVA, permutational multivariate analysis of variance, RA, retinoic acid, RBP or RBP4, retinol-binding protein, *Rbp*^*−/−*^, retinol-binding protein–deficient mice, RE, retinyl esters, *RegIII*, regenerating islet-derived protein 3, ROH, retinol, ROS, reactive oxygen species, SCFA, short-chain fatty acid, VA, vitamin A, VAD, vitamin A deficiency, VA-def, vitamin A deficient, VA-suf, vitamin A sufficient

## Abstract

Lecithin:retinol acyltransferase and retinol-binding protein enable vitamin A (VA) storage and transport, respectively, maintaining tissue homeostasis of retinoids (VA derivatives). The precarious VA status of the lecithin:retinol acyltransferase–deficient (*Lrat*^−/−^) retinol-binding protein–deficient (*Rbp*^*−/−*^) mice rapidly deteriorates upon dietary VA restriction, leading to signs of severe vitamin A deficiency (VAD). As retinoids impact gut morphology and functions, VAD is often linked to intestinal pathological conditions and microbial dysbiosis. Thus, we investigated the contribution of VA storage and transport to intestinal retinoid homeostasis and functionalities. We showed the occurrence of intestinal VAD in *Lrat*^*−/−*^*Rbp*^*−/−*^ mice, demonstrating the critical role of both pathways in preserving gut retinoid homeostasis. Moreover, in the mutant colon, VAD resulted in a compromised intestinal barrier as manifested by reduced mucins and antimicrobial defense, leaky gut, increased inflammation and oxidative stress, and altered mucosal immunocytokine profiles. These perturbations were accompanied by fecal dysbiosis, revealing that the VA status (sufficient vs. deficient), rather than the amount of dietary VA per se, is likely a major initial discriminant of the intestinal microbiome. Our data also pointed to a specific fecal taxonomic profile and distinct microbial functionalities associated with VAD. Overall, our findings revealed the suitability of the *Lrat*^*−/−*^*Rbp*^*−/−*^ mice as a model to study intestinal dysfunctions and dysbiosis promoted by changes in tissue retinoid homeostasis induced by the host VA status and/or intake.

The essential nutrient vitamin A (VA) is critical for maintaining a plethora of mammalian biological functions, mainly because of the action of its active form, retinoic acid (RA), that controls gene transcription via binding to RA receptors and retinoid X receptors (1, 2). Alterations of tissue concentrations of retinoids (VA and its derivatives) have been linked to chronic diseases in humans, including metabolic (3), respiratory (4), and intestinal disorders (5).

Tissue retinoid homeostasis is maintained by a fine balance between VA transport to the target organs and conversion to VA ester reserves to be used in times of need. In the fasting state, the delivery of VA to extrahepatic organs is achieved predominantly via retinol (ROH) bound to retinol-binding protein (RBP or RBP4) upon its secretion from the liver, the major body reserve of retinoids (6). After a meal, however, up to 25% of the dietary vitamin can be distributed to the extrahepatic tissues packaged within chylomicron remnants, generated upon LPL-mediated remodeling of the chylomicrons synthesized by the enterocytes (7). Formation of retinyl esters (REs), the storage form of VA, occurs through esterification of ROH primarily by the action of the enzyme lecithin:retinol acyltransferase (LRAT), which is widely expressed in tissues (7). The critical role of RBP and LRAT in maintaining tissue retinoid homeostasis in adult and developing tissues has been confirmed mainly by investigating the consequences of the lack of *Rbp* and/or *Lrat* in genetically modified mouse models (6, 8, 9, 10, 11, 12, 13).

In the intestine, VA modulates morphology and functions by maintaining epithelial barrier integrity and regulating gut immunity and inflammation (14, 15). Not surprisingly, VA deficiency (VAD) is often associated with pathological conditions of the gut (5). Solely gauged on circulating levels of ROH (VA alcohol) (16), VAD is still one of the most widespread micronutrient deficiencies (17). Often linked to general malnutrition in developing countries (17), VAD is also emerging in industrialized nations as a health issue affecting mostly people of low socioeconomic backgrounds who consume diets particularly low in fruits and vegetables but not necessarily in calories (18, 19, 20). Importantly, a role of VA as a modulator of the microbiome is also becoming apparent. For instance, VAD has been linked to fewer butyrate-producing *Clostridia sp.* in the feces of both children (21) and mice (22). Furthermore, a few bacterial species have been reported to be sensitive to the concentration of VA (ROH) in the diet in a gnotobiotic mouse model (23). More recently, specific gut bacteria within the class *Clostridia* have been shown to prevent microbial dysbiosis by regulating RA metabolism in intestinal epithelial cells (24). The intestine is the only organ in the body directly exposed to dietary VA. Yet, the impact of the pathways of VA transport and stores formation versus the dietary VA per se in maintaining intestinal retinoid homeostasis and functions is unknown.

Here, we used a genetic model of VAD, the lecithin:retinol acyltransferase–deficient (*Lrat*^*−/−*^) retinol-binding protein–deficient (*Rbp*^*−/−*^) mice (11, 25). Unable to store VA (because of the lack of LRAT) and to efficiently mobilize hepatic ROH toward the peripheral tissues (because of the lack of RBP), these mutant mice rely solely on dietary intake to support VA-dependent functions. When fed diets contain retinoids, these mutant mice are viable and fertile without showing obvious phenotypes (11, 25). However, their precarious VA status rapidly deteriorates upon dietary VA deprivation, leading to signs of severe VAD (11, 25). The present results show that dual ablation of *Lrat* and *Rbp*, coupled with dietary VA restriction, rendered the intestine of the mutant mice susceptible to developing retinoid deficiency, which was accompanied by fecal dysbiosis and dysfunctions in the colon, where the majority of the microbial communities reside. This model also enabled us to evaluate the role of the host VA status and intake in driving taxonomic and functional changes in the fecal microbiome.

## Materials and methods

### Mice and nutritional manipulation

WT and *Lrat*^*−/−*^*Rbp*^*−/−*^ mice on a mixed genetic background (C57BL/6J × 129/Sv; (11)) were maintained on a standard VA-sufficient (VA-suf) chow diet containing 18 IU VA/g diet (Prolab Isopro RMH 3000 5p75). At the beginning of this study, double-heterozygous mice (*Lrat*^*+/−*^*Rbp*^*+/−*^) were crossed to obtain F1 WT (*Lrat*^*+/+*^*Rbp*^*+/+*^) and double-KO (*Lrat*^*−/−*^*Rbp*^*−/−*^) progenies (at the expected Mendelian ratio). From this F1, WT and *Lrat*^*−/−*^*Rbp*^*−/−*^ were each maintained as an inbred line through parallel breeding. The genetic background of these two lines was tested with a Mouse 384 SNP panel (Jackson Laboratories, Bar Harbor, ME) to determine the percentage of the C57BL/6J and 129/Sv genetic background, which was about 50:50 in both lines. At six weeks of age, *Lrat*^*−/−*^*Rbp*^*−/−*^ and WT female mice, raised on the above-described regular chow diet, were placed either on a purified VA-suf (Research Diets; 20 IU VA/g diet) or VA–deficient (VA-def) diet (Research Diets: < 0.02 IU VA/g diet) for four weeks. The macronutrient composition of these diets was similar (protein 26%, carbohydrate 60%, fat 14% Kcal). At ten weeks of age, after collection of fresh feces, mice (n = 3–6/group) were sacrificed by CO_2_ inhalation between 9:30 and 11:30 AM. The serum, liver, adipose tissue (perigonadal), small intestine (duodenum mucosa), and colon were collected, immediately snap-frozen on dry ice, and stored at −80 °C for microbiome DNA and HPLC analyses. To carry out these experiments, 2–3 mice of the same genotype were housed per cage. At dissection, the duodenum (the proximal one-third of the small intestine) was opened longitudinally, food content was removed, and the mucosa was gently scraped. The colon was also opened longitudinally to remove feces and food contents, but this segment of the intestine was collected as is, that is, without scraping the mucosa. Note that the terms small intestine and duodenum will be used interchangeably throughout the text and both refer to scraped mucosa. As colon and duodenum mucosa from each mouse was used mostly for two types of analysis, additional mice underwent the same dietary regimen described above and were sacrificed after four weeks on the above-described purified diets to perform the following analysis: n = 6/group for mRNA analysis; n = 3/group for Periodic Acid-Schiff (PAS) staining of the intestine; n = 4–5/group for in vivo reactive oxygen species (ROS) measurement; n = 4–6 for LC-MS/MS measurements of tissue RA concentration. After four weeks on the diets described above, another subset of *Lrat*^*−/−*^*Rbp*^*−/−*^ mice (n = 3/group) was placed on the VA-suf diet for one week before sacrifice, when feces and tissues were collected as described above.

Throughout the experiment, mice had constant access to diet and water ad libitum and were housed in a room with a temperature of 24 ± 1°C and a 12:12-h light:dark cycle (7:00 AM–7:00 PM). Food intake, measured weekly from six to ten weeks of age, was similar among the groups (Fig. S1A). *Lrat*^*−/−*^*Rbp*^*−/−*^ mice showed a slightly but significantly lower body weight than WT mice fed the VA-suf diet throughout the experiment, regardless of the dietary regimen (Fig. S1B). All animal experiments were conducted in accordance with the National Institutes of Health Guide for the Care and Use of Laboratory Animals and were approved by the Rutgers University Institutional Committee on Animal Care.

### HPLC and LC-MS/MS analysis of retinoids

Reversed-phase HPLC analyses of ROH and RE concentrations in serum and tissues were performed as described previously (26). For the small intestine and colon, 100 to 150 mg tissue were used. RA in serum and tissues was measured by LC-MS/MS as described previously (27). In brief, tissues (100 to 120 mg liver or 120 to 150 mg small intestine or colon) were homogenized with 0.9% saline and 100 μl serum was diluted with saline, both in a 5:1 ratio of saline-to-tissue homogenate or serum. After the addition of internal standard [5 μl of 2 μM all-*trans*-RA-d_5_; Toronto Research Chemicals (North York, Ontario, Canada)], 2 ml of acetonitrile with 1% formic acid was added to tissue homogenates and diluted serum followed by the addition of 10 ml of hexanes to extract different RA isomers (all-*trans*-, 13-*cis*-, and 9-*cis*-RA). After the organic layer was transferred to a new glass tube and evaporated under N_2_ flow, samples were reconstituted in 60% acetonitrile and analyzed using the AB Sciex 5500 QTRAP Mass Spectrometer coupled with an Agilent 1290 UHPLC (Agilent Technologies, Santa Clara, CA). The LC-MS/MS method used for the analysis of RA including LC conditions and MS parameter settings, was as reported previously (28).

### RNA extraction and quantitative real-time PCR

RNA extraction and quantitative real-time PCR were performed as described previously (29). Briefly, total mRNA was extracted from mouse colon using RNA-Bee according to the manufacturer's instructions (Tel-test Inc., Friendswood, TX). The concentration and purity of RNA were determined by Nanodrop 2000 Spectrophotometer (Thermo Fisher Scientific, Waltham, MA). One microgram of RNA was reverse-transcribed to complementary DNA using Verso cDNA synthesis kit according to the manufacturer's instructions (Thermo Fisher Scientific, Dallas, TX). To quantify mRNA, real-time PCR was performed using an Applied Biosystems QuantStudio 3 Applied Biosystem instrument (Thermo Fisher Scientific, Dallas, TX). For the qPCR experiments, 300 nM of each specific primer (listed in Table S1) was mixed with 25–50 ng of cDNA equivalent of the total RNA, and 7.5 μl of SYBR Green Master Mix (Thermo Fisher Scientific, Dallas, TX) in a total volume of 15 μl. Each sample was run in duplicates or triplicates. Relative quantification of mRNA expression was calculated using 2(−ΔΔCt) method (30), normalized to the TATA-binding protein gene. Gene expression changes were expressed as mRNA fold change from the control group (WT on VA-suf).

### PAS/Alcian blue analysis

The entire colon segment of the gastrointestinal tract was dissected and fixed overnight at room temperature in 10% formalin. For each mouse, six 2.5 mm segments were collected every 10 mm along the length of the colon and randomly embedded in the same paraffin block. For each paraffin block, 5 μm sections were cut at three different depths (levels), at an average of 400 μm apart. At each level, one section was collected and stained with the Richard-Allen Scientific Periodic Acid-Schiff kit, which stains acidic and neutral mucins (*Muc*) for goblet cell visualization and quantification (31). All tissue sections were scanned using a Revolve microscope (ECHO Laboratories, San Diego, CA).

### FITC-dextran intestinal permeability assay

Intestinal permeability was assessed as described by Lackey *et al.* (32). After a 6-h fast, mice were orally gavaged with 100 μl of FITC-dextran (MW = 4,000; 150 mg/ml; catalog no. 60842-46-8, Millipore Sigma). Four hours after gavage, mice were euthanized and whole blood was collected. Serum was isolated by centrifugation and kept in the dark at room temperature for 30 min before analysis. A standard curve was developed on a 96-well plate using FITC-dextran concentrations in a range between 100 and 1 μg/ml. Fluorescence intensities of the standards and samples were then measured using an excitation of 485 nm and emission of 528 nm. After blank subtraction, sample FITC concentrations were determined based on the standard curve (in μg/ml).

### Preparation of hydro-indocyanine green and in vivo ROS imaging and analysis

Hydro-indocyanine green was prepared from the cyanine dye, indocyanine green, by reduction with NaBH_4_ as described previously (33). WT and *Lrat*^*−/−*^*Rbp*^*−/−*^ mice at ten weeks of age were gavaged with hydro-indocyanine green reconstituted in water (0.5 mg/ml) at a dose of 2 mg/kg. In vivo ROS imaging and analysis was performed by using a Bruker In-Vivo Multispectral FX PRO imaging system (Bruker, Ettlingen, Germany). Briefly, after mice were anesthetized with 2% isoflurane, they were laid down in the abdomen-down position directed toward the camera. After excitation illumination at 760 nm, emission at 830 nm was recorded using a filter equipped high-sensitivity cooled charge-coupled device camera. Acquisition time was 30 s for near-infrared fluorescence (NIRF) images, followed by a bright-field light photograph (0.5 s exposure). Both NIRF and bright-field images were optically superimposed to visualize anatomical information. Fluorescence was quantified as photons/s/mm^2^ using Carestream MI software v5/0.529 (Carestream Health Inc., Rochester, NY). The background intensity of each image was set to zero and identical elliptical regions of interest were drawn on each image (91 × 103 pixels; interior area = 7,373). The mean fluorescence intensity within the ellipse was recorded for each animal.

### Short-chain fatty acids measurements by GC-MS analysis

Freshly collected fecal samples were weighed and resuspended in 1 ml of 0.5% phosphoric acid per 0.1 g of sample and frozen at −20°C immediately after collection. Once thawed, the fecal suspensions were homogenized for about 2 min and centrifuged for 10 min at 17,949 *g*. The aqueous fecal suspensions were extracted with diethyl ether (1:1, vol:vol) by vortexing for 30 s followed by centrifugation for 10 min at 17,949 *g*. Organic extracts were stored at −20°C. Before analysis, a 600 μl volume of the organic phase was transferred into a tube and 100 μl of 1 mM 2-methyl hexanoic acid (Thermo Fisher Scientific, Dallas, TX) was added as internal standard. The internal standard was used to correct for injection variability between samples and minor changes in the instrument response. Three independent replicate extractions were performed per sample.

The GC-MS analysis was performed as described (34). Briefly, the GC-MS system consisted of an Agilent 7890A (Agilent Technologies, Palo Alto, CA), equipped with an automatic liquid sampler (MPS2) (Gerstel, Mulheim, Germany) and coupled to an Agilent 5975C mass selective detector. The GC was fitted with a high polarity, polyethylene glycol, fused silica capillary column DB-WAXetr (30 m, 0.25 mm id, 0.25 μm film thickness) and helium was used as the carrier gas at 1 ml/min. Injection was made in a splitless mode with an injection volume of 1 μl and an injector temperature of 250°C. Data acquisition was performed using the ChemStation software (Hewlett-Packard, Palo Alto, CA). Identification of the short-chain fatty acids (SCFAs) was based on the retention time of standard compounds and with the assistance of the NIST 08 libraries. SCFA absolute concentrations were determined based upon standard curves generated for each individual fatty acid.

### Fecal sample collection and DNA extraction

Each mouse was placed in an empty cage without bedding for 10–15 min to allow collection of fresh stool samples that were snap-frozen in dry ice and kept at -80°C until further processing. Fecal genomic DNA was extracted at the University of Missouri DNA Core facility using PowerFecal kits (Qiagen, Hilden, Germany) according to the manufacturer's instructions, with the exception that the samples were homogenized in the provided bead tubes using a TissueLyser II (Qiagen, Venlo, the Netherlands) for three minutes. DNA yields were quantified via fluorometry (Qubit 2.0, Invitrogen, Carlsbad, CA) using quant-iT BR dsDNA reagent kits (Invitrogen).

### 16S rRNA library construction and sequencing

Library construction and sequencing were performed at the University of Missouri DNA Core facility (35). Bacterial 16S rRNA amplicons were generated via amplification of the V4 hypervariable region of the 16S rRNA gene using dual-indexed universal primers (U515F/806R) flanked by Illumina standard adapter sequences and the following parameters: 98°C(3:00) + [98°C(0:15) + 50°C(0:30) + 72°C(0:30)] × 25 cycles + 72°C(7:00). PCR was performed in 50 μl reactions containing 100 ng DNA, primers (0.2 μM each), dNTPs (200 μM each), and Phusion high-fidelity DNA polymerase (1 U; Thermo Fisher Scientific, Waltham, MA). Amplicon pools (5 μl/reaction) were combined, thoroughly mixed, and then purified by addition of Axygen AxyPrep MAG PCR clean-up beads (Thermo Fisher Scientific, Waltham, MA) to an equal volume of 50 μl of amplicons and incubated for 15 min at room temperature. Products were then washed multiple times with 80% ethanol, and the dried pellet was resuspended in 32.5 μl of elution buffer, incubated for 2 min at room temperature, and then placed on the magnetic stand for 5 min. The final amplicon pool was evaluated using the Advanced Analytical Fragment Analyzer automated electrophoresis system (Agilent, Santa Carla, CA), quantified using quant-iT HS dsDNA reagent kits (Invitrogen), and diluted according to Illumina's standard protocol for sequencing on the MiSeq instrument (Illumina, San Diego, CA), using the V2 chemistry to generate 2 × 250 bp paired-end reads.

### Informatics analysis

Read merging, clustering, and annotation of DNA sequences were performed at the University of Missouri Informatics Research Core Facility. Paired DNA sequences were merged using FLASH software and removed if found to be far from the expected length of 292 bases after trimming for base quality of 31. Cutadapt (https://github.com/marcelm/cutadapt) was used to remove the primers at both ends of the contig and cull contigs that did not contain both primers. The u-search fastq_filter command (http://drive5.com/usearch/manual/cmd_fastq_filter.html) was used for quality trimming of contigs, rejecting those for which the expected number of errors was greater than 0.5. All contigs were trimmed to 248 bases, and shorter contigs were removed. The Qiime (36) 1.9 command split_libraries_fastq.py was used to demultiplex the samples, and the command beta_diversity_through_plots.py was used to subsample data to a uniform read count. The outputs for all samples were combined into a single file for clustering. The UPARSE method (http://www.drive5.com/uparse/) was used to both clusters contigs with 97% identity and remove chimeras. Taxonomy was assigned to selected operational taxonomic units (OTUs) using BLAST (37) against the SILVA database v132 (38) of 16S rRNA gene sequences and taxonomy.

The 16S rRNA–derived OTUs were mapped to fusionDB (39). The microbial functionalities of the mouse fecal microbiome samples were assessed by the pan-functional-repertoire of each OTU (i.e., the functions that exist in at least one member of bacterial corresponding OTU). Then, nonmetric multidimensional scaling analysis and the subsequent permutational multivariate analysis of variance (PERMANOVA) test using the Vegan R package were performed.

### Statistical analysis

SPSS statistical software (IBM SPSS Statistics, version 23; SPSS, Inc.) was used for statistical analysis. The distribution of the data was determined by the Shapiro-Wilk test, and normally distributed data were analyzed by Student's *t*-test or a two-way ANOVA. Non-normally distributed data were analyzed by the Mann-Whitney *U* test or a Kruskal-Wallis test. Values are presented as the mean ± SD, and *P* < 0.05 was the cutoff for significance.

## Results

### *Lrat*^*−/−*^*Rbp*^*−/−*^ mice as a model of VAD

**Fig. 1:**
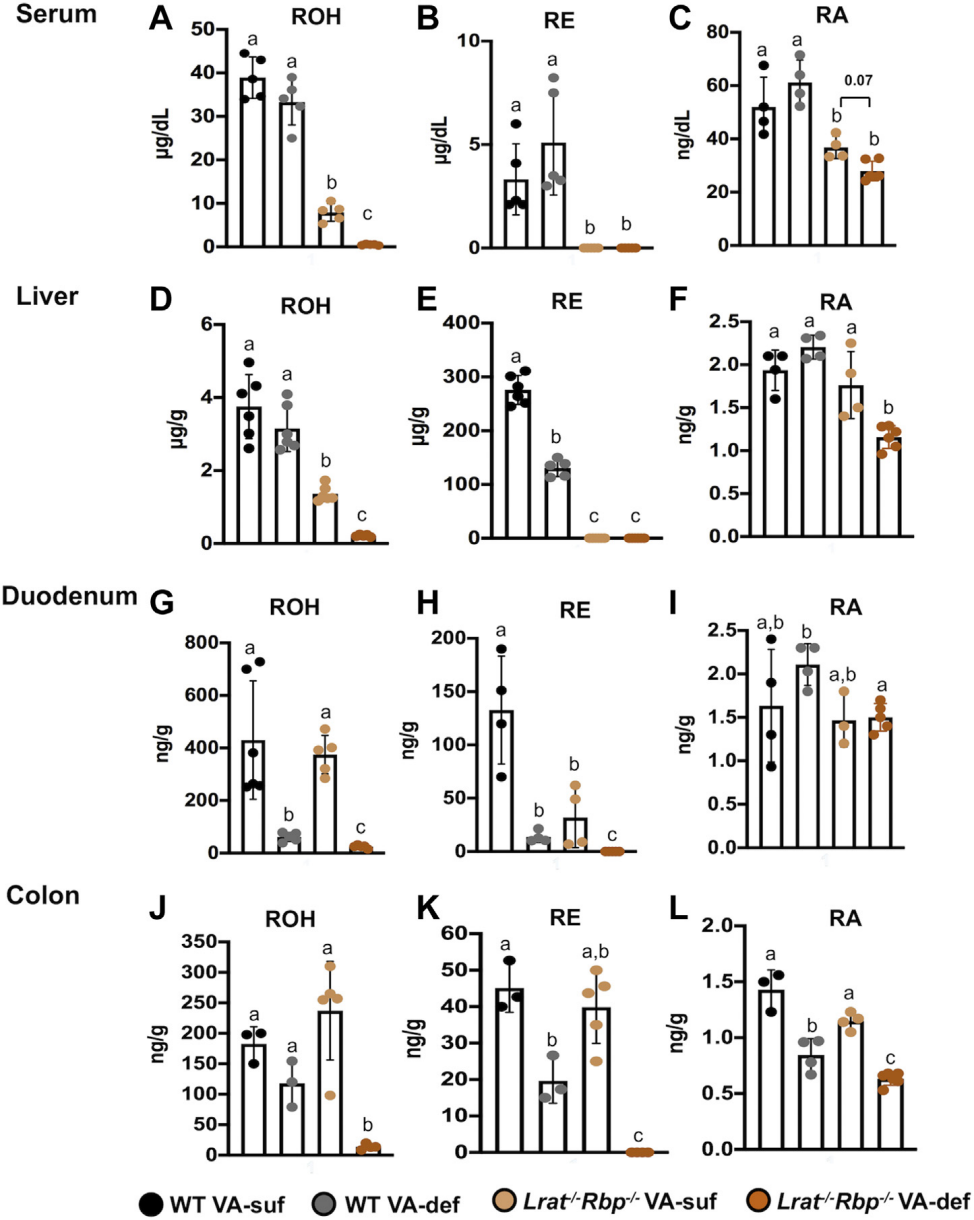
Serum and tissue retinoid concentrations in *Lrat*^*−/−*^*Rbp*^*−/−*^ and WT mice. Serum (A) retinol (ROH), (B) retinyl ester (RE), and (C) retinoic acid (RA); liver (D) retinol (ROH), (E) retinyl ester (RE), and (F) retinoic acid (RA); duodenum (G) retinol (ROH), (H) retinyl ester (RE), and (I) retinoic acid (RA); colon (J) retinol (ROH), (K) retinyl ester (RE), and (L) retinoic acid (RA) were determined by reversed-phase HPLC (retinol and retinyl ester) and LC-MS/MS (retinoic acid). Data are the mean ± SD; n = 3–6 mice/group. Statistical analysis by two-way ANOVA for normally distributed data and by the Mann-Whitney test for non-normally distributed data. Different letters indicate significant differences (*P* < 0.05) among the groups. *Lrat*^−/−^, lecithin:retinol acyltransferase–deficient; *Rbp*^*−/−*^, retinol-binding protein–deficient; VA-def, vitamin A deficient; VA-suf, vitamin A sufficient.

At six weeks of age, *Lrat*^*−/−*^*Rbp*^*−/−*^ and WT mice were placed on diets with or without VA for four weeks, when they were sacrificed to collect serum and tissues (see Materials and Methods). We first measured retinoid levels in the serum and liver by HPLC (26) and LC-MS/MS (27, 28). Owing to the absence of RBP (6, 11), serum ROH levels in *Lrat*^*−/−*^*Rbp*^*−/−*^ mice were reduced ∼80% on the VA-suf and ∼98% on the VA-def compared with WT mice on the same dietary regimen (Fig. 1A). In contrast but as expected (6, 11, 12), under this VA-def regimen, ROH levels in WT mice remained steady (Fig. 1A). Ablation of LRAT (8, 9, 10) rendered serum REs undetectable in *Lrat*^*−/−*^*Rbp*^*−/−*^ mice, regardless of the dietary regimen (Fig. 1B). RA levels were also attenuated in *Lrat*^*−/−*^*Rbp*^*−/−*^ mice compared with the WT groups on both diets (Fig. 1C). Moreover, RA concentration showed a trend (*P* = 0.07) toward a reduction in the *Lrat*^*−/−*^*Rbp*^*−/−*^ VA-suf versus the *Lrat*^*−/−*^*Rbp*^*−/−*^ VA-def group (Fig. 1C). In contrast, dietary VA restriction did not attenuate serum RA concentration in the WT mice (Fig. 1C).

The hepatic ROH concentration was significantly lower in the *Lrat*^*−/−*^*Rbp*^*−/−*^ mice than in the WT regardless of the dietary VA regimen (Fig. 1D), and REs were undetectable (Fig. 1E), as reported previously (11). Liver ROH (Fig. 1D) and RA concentration (Fig. 1F) in the *Lrat*^*−/−*^*Rbp*^*−/−*^ mice further declined upon dietary VA deprivation, whereas in WT mice, four weeks of VA-def diet decreased hepatic RE by 50% (Fig. 1E) but not ROH (Fig. 1D) or RA levels (Fig. 1F). Overall, owing to the genetic manipulation, hepatic total ROH (ROH + REs) levels were dramatically depleted in *Lrat*^*−/−*^*Rbp*^*−/−*^ mice already on the VA-suf diet, whereas a 40% reduction in RA levels developed in the mutant livers only under the VA-def diet. Previous research pointed to a compensatory mechanism that enables mice to build retinoid stores in the adipose tissue in the absence of LRAT (13). Indeed, compared with WT mice, ROH (Fig. S2A) and RE (Fig. S2B) concentrations were elevated in the adipose tissue of the *Lrat*^*−/−*^*Rbp*^*−/−*^ mice maintained on the VA-suf diet. Moreover, dietary VA deprivation depleted adipose ROH and RE stores in the mutants but only retinol in WT mice (Fig. S2A, B). Thus, liver and adipose retinoid concentrations confirmed that *Lrat*^*−/−*^*Rbp*^*−/−*^ mice have very limited retinoid reserves already when fed the VA-suf diet and that the lack of dietary VA nearly exhausted these stores and reduced hepatic RA levels. This is consistent with what appears to be a more severe VAD of the mutant mice on the VA-def than VA-suf diet, based on their serum retinoid levels.

Taken together, these data confirmed the precarious VA status of the double-KO mice and their sensitivity to dietary VA insufficiency.

### VA homeostasis in the gastrointestinal tract of the *Lrat*^*−/−*^*Rbp*^*−/−*^ mice

VA homeostasis in the gastrointestinal tract of the *Lrat*^*−/−*^*Rbp*^*−/−*^ strain was never investigated before. We measured retinoid levels by HPLC (26) and LC-MS/MS (27, 28) in the small intestine and colon of these mice. In the small intestine (duodenum), ROH levels were similar between genotypes on the VA-suf diet (Fig. 1G). Lack of dietary VA significantly diminished the duodenum ROH content in both genotypes with an additional significant reduction in *Lrat*^*−/−*^*Rbp*^*−/−*^ versus WT mice (Fig. 1G). REs were also significantly diminished in the small intestine of the *Lrat*^*−/−*^*Rbp*^*−/−*^ mice compared with the WT on the VA-suf diet (Fig. 1H), confirming that LRAT is the main enzyme that esterifies ROH in the duodenum (10, 40). However, RE levels dropped significantly also in the WT duodenum upon dietary VA restriction, pointing to the dietary origin of the majority of the retinoids in this segment of the intestine (Fig. 1H). Nevertheless, RA concentrations remained steady in the duodenum of the WT mice, whereas it was significantly reduced in the duodenum of the *Lrat*^*−/−*^*Rbp*^*−/−*^ mice on the VA-def diet compared with the WT on the same dietary regimen (Fig. 1I). Surprisingly, in the colon, ROH (Fig. 1J) and REs (Fig. 1K) levels were similar between genotypes on the VA-suf diet. Notably, dietary VA deprivation significantly reduced colon ROH in *Lrat*^*−/−*^*Rbp*^*−/−*^ mice (Fig. 1J), and RE levels in both strains, although this latter reduction was more dramatic in the mutants (Fig. 1K). Colon RA levels were also similar between the two genotypes on the VA-suf diet (Fig. 1L), but the lack of dietary VA significantly diminished RA in both WT and *Lrat*^*−/−*^*Rbp*^*−/−*^ mice, compared with their respective genotype group on the VA-suf diet. Notably, the VA-def mutants displayed a significantly lower colon RA concentration than the WT on VA-def diets (Fig. 1L).

These data demonstrated the precarious retinoid status of the intestine of the mice lacking *Rbp* and *Lrat*, which was exacerbated secondary to the dietary VA restriction in both segments of the gut. Surprisingly, the lack of dietary VA attenuated RA concentration in the colon, but not the duodenum, of the WT mice.

### The taxonomic profile of the fecal microbiome is altered in *Lrat*^*−/−*^*Rbp*^*−/−*^ mice

**Fig. 2:**
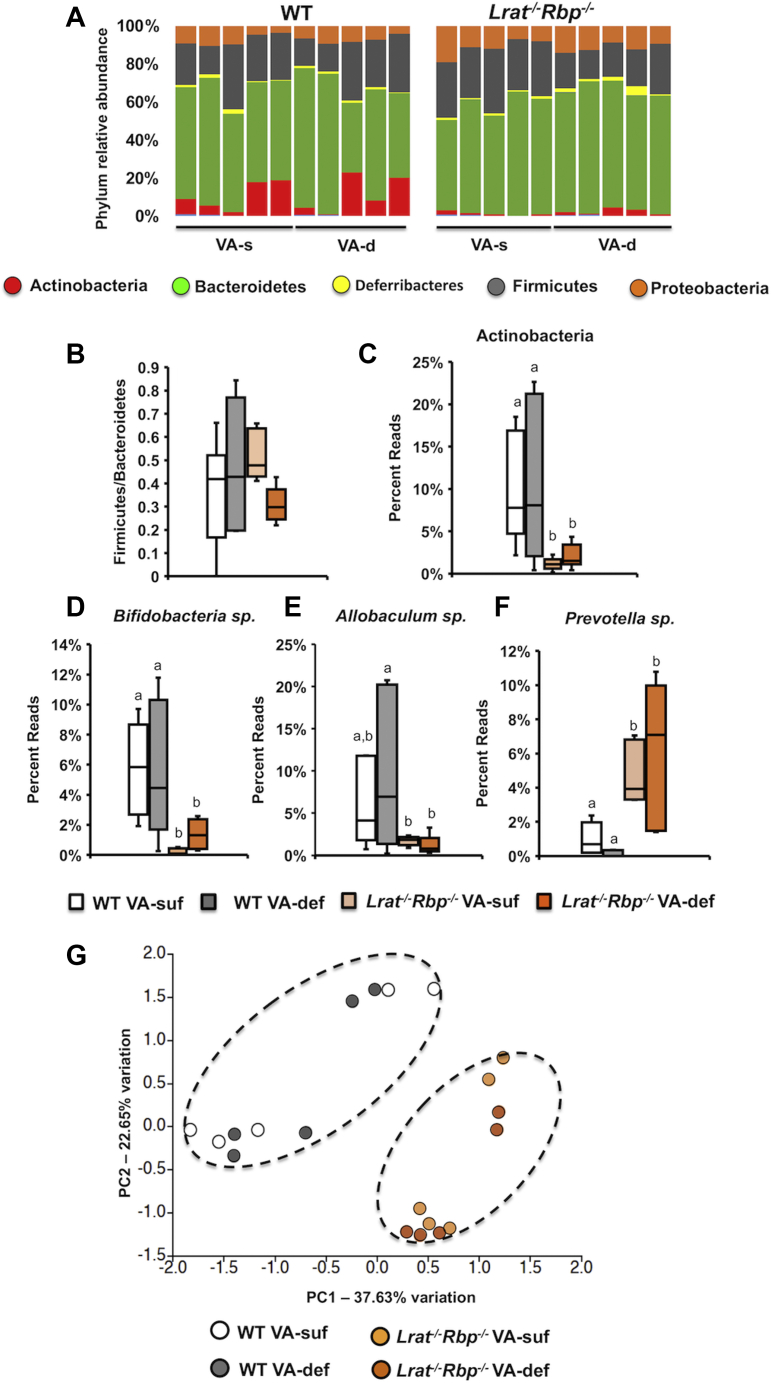
Fecal microbial composition in *Lrat*^*−/−*^*Rbp*^*−/−*^ and WT mice. A: Relative abundance of bacterial phyla in mouse fecal samples. Percent ratio reads of (B) Firmicutes/Bacteroidetes, (C) Actinobacteria, (D) *Bifidobacteria sp.*, (E) *Allobaculum sp.,* and (F) *Prevotella sp.* in the fecal microbiota. Statistical analysis by two-way ANOVA. Different letters indicate significant difference (*P* < 0.05) among the groups. G: Principal component analysis (PCA) of the 16S rRNA genes in fecal samples. PC1 separated samples by genotype, that is, vitamin A status and accounted for 37.63% of total variance; n = 5 mice/group. *Lrat*^−/−^, lecithin:retinol acyltransferase–deficient; *Rbp*^*−/−*^, retinol-binding protein–deficient. VA-d or VA-def, vitamin A deficient diet; VA-s or VA-suf, vitamin A sufficient diet.

**Fig. 3:**
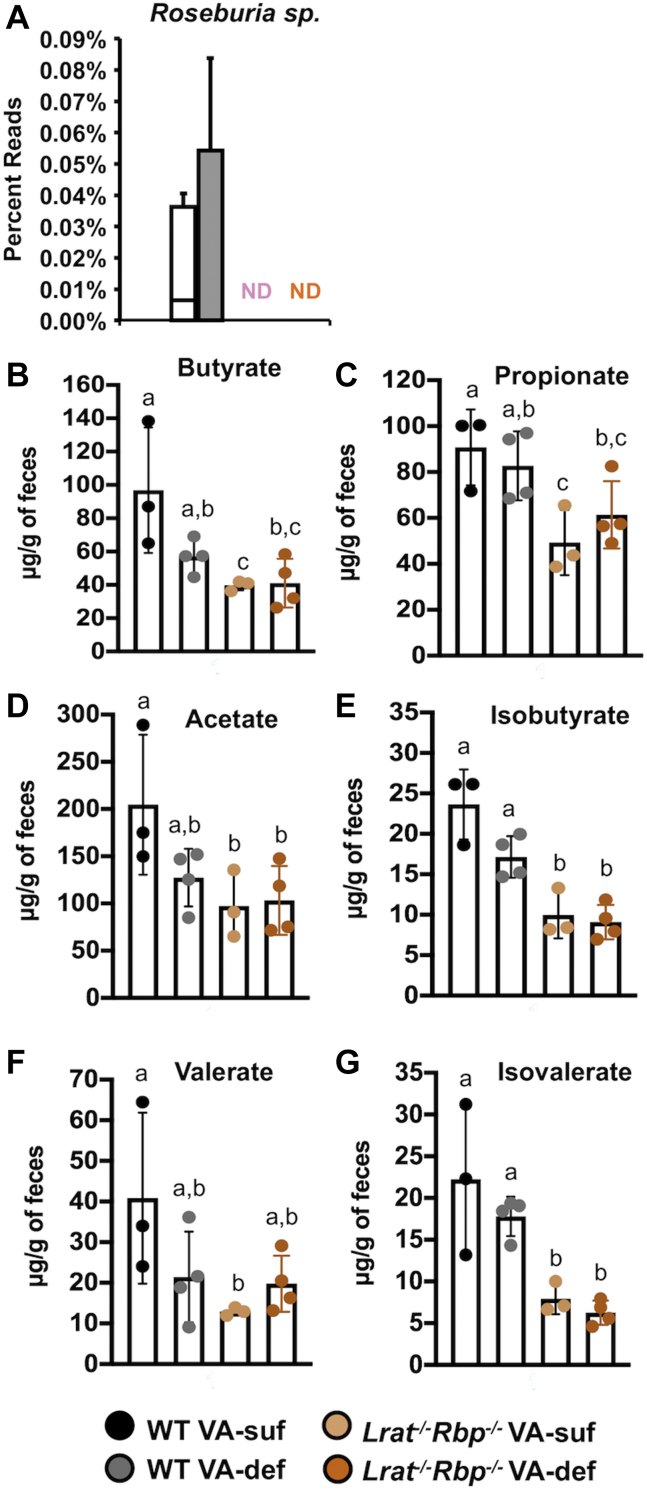
Vitamin A deficiency in *Lrat*^*−/−*^*Rbp*^*−/−*^ and WT mice reduces butyrate production. A: Percent genus reads of *Roseburia* sp. in the fecal microbiota of the four groups of mice (white bar indicate WT VA-suf; gray bar indicates WT VA-def). B–G: The concentration (μg/g wet feces) of the short-chain fatty acids (SCFAs) in mouse fecal samples. Values are the mean ± SD; n = 3–4 samples/group; statistical analysis two-way ANOVA. Different letters indicate significant differences among the groups (*P* < 0.05). *Lrat*^−/−^, lecithin:retinol acyltransferase–deficient; ND, not detected; *Rbp*^*−/−*^, retinol-binding protein–deficient; VA-def, vitamin A deficient; VA-suf, vitamin A sufficient.

We next asked whether the altered retinoid homeostasis in the intestine of the mutant mice corresponded to changes in the fecal microbial community. To this end, DNA was extracted from the feces to sequence the 16S rRNA genes and annotate them against the SILVA database of 16S rRNA gene sequences (38). α-Diversity, assessed based on Shannon, Simpson, and Chao-1 indexes, was similar among the experimental groups, regardless of the genotype and/or diet (Table S2). Annotation of sequence data to the level of phyla revealed *Actinobacteria*, *Bacteroidetes*, *Deferribacteres*, *Firmicutes*, and *Proteobacteria* as the most abundant phyla in the fecal microbial population, regardless of the genotype and/or diet (Fig. 2A), as reported in mice and humans (41). The ratio between *Firmicutes* and *Bacteroidetes* was not significantly different among the experimental groups (Fig. 2B). By contrast, *Actinobacteria* were significantly reduced in the *Lrat*^*−/−*^*Rbp*^*−/−*^ mice compared with the WT groups on both diets (Fig. 2A, C). Furthermore, a significant reduction of *Bifidobacterium* sp. (phylum of *Actinobacteria*) and *Allobaculum* sp. (phylum of Firmicutes), as well as a higher abundance of the *Prevotella* sp. (phylum of *Bacteroidetes*) were observed in the *Lrat*^*−/−*^*Rbp*^*−/−*^ mice than WT groups on both diets (Fig. 2D–F). Further differences in community composition (i.e., β-diversity) among the four groups were analyzed by principal component analysis (PCA). Figure 2G shows that the samples clustered based on the genotype (PC1, accounting for 37.63% of variance) and not on the VA content of the diet. Upon generating a loading plot from the same dataset used for the PCA, we determined which OTUs were the major contributors to the differences associated with the genotype. As shown in Table 1, the mutants showed a higher abundance of bacteria such as *Bacteroides* spp., *Sutterella*, *Helicobacter apodemus*, and *Paraprevotella* spp., and the family *Mogibacteriaceae*, whereas the WT displayed greater abundance of *Allobaculum* spp, *Bifidobacterium*, and *Rikenella* spp., as well as of the family *Coriobacteriaceae*. Moreover, based on 16S rRNA gene sequence analysis, butyrate-producing bacteria, such as *Roseburia* sp., were undetectable in the feces of the *Lrat*^*−/−*^*Rbp*^*−/−*^ mice, regardless of their dietary intake of the vitamin (Fig. 3A), whereas *Clostridium* sp. were undetectable exclusively in fecal samples of the *Lrat*^*−/−*^*Rbp*^*−/−*^ VA-suf group (data not shown). In agreement with these findings, direct measurements of SCFAs in fecal samples confirmed significantly lower levels of all SCFA, including butyrate, in the feces of the *Lrat*^*−/−*^*Rbp*^*−/−*^ mice, whereas the dietary VA intake per se did not affect the SCFA concentration, at least in the feces, regardless of the genotype (Fig. 3B–G). Taken together these data showed fecal dysbiosis in the *Lrat*^*−/−*^*Rbp*^*−/−*^ mice at the level of bacterial taxa that is not substantially modified by the dietary VA level.

**Table 1:** Relative abundance of bacterial OTUs in WT and *Lrat*^*−/−*^*Rbp*^*−/−*^ fecal samples

Higher Relative Abundance in WT on VA-suf versus VA-def		Higher Relative Abundance in *Lrat*^*−/−*^*Rbp*^*−/−*^ on VA-suf versus VA-def	
*Bifidobacterium* sp.	*P* = 0.005	*Oscillospira* sp.	*P* < 0.001
*Allobaculum* sp.	*P* < 0.05	*Bacteroides* sp.	*P* < 0.05
*Rikenella* sp.	*P* < 0.05	*Helicobacter apodemus*	*P* < 0.005
*Mucispirillum* sp.	*P* < 0.05	*Sutterella* sp.	*P* < 0.005
*Desulfovibrio* C21_c20	*P* < 0.05	*Paraprevotella* sp.	*P* < 0.005
*Roseburia* sp.	*P* < 0.05	*Prevotella* sp.	*P* < 0.05
*Flexispira* sp.	*P* < 0.05	*Mycoplasma muris*	*P* < 0.005
UC *Mycoplasmataceae*	*P* < 0.05	*Coprococcus* sp.	*P* < 0.005
UC *Coriobacteriaceae*	*P* < 0.05	UC *Desulfovibrionaceae*	*P* < 0.005
Order *Rickettsiales*	*P* < 0.05	UC *Mogibacteriaceae*	*P* < 0.001
		UC *Christensenellaceae*	*P* < 0.005
		UC *Erysipelotrichaceae*	*P* < 0.05
		UC *Ruminococcaceae*	*P* < 0.05
		UC *Peptococcaceae*	*P* < 0.05

Statistical analysis of the fecal bacterial OTU abundance between WT and *Lrat*^*−/−*^*Rbp*^*−/−*^ strain by the *t*-test.

OTUs, operational taxonomic units; VA-def, vitamin A–deficient diet; VA-suf, vitamin A–sufficient diet.

### VA dietary restriction affects fecal microbial functionalities in the *Lrat*^*−/−*^*Rbp*^*−/−*^ mice

**Fig. 4:**
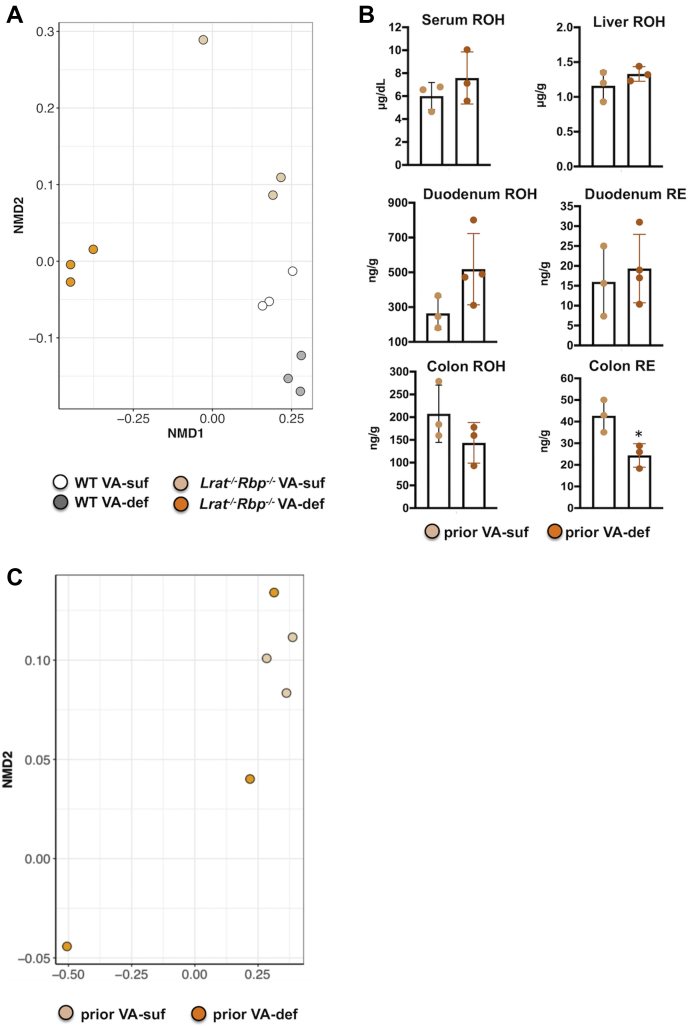
FusionDB-derived microbiome functional profile parallels vitamin A status. A: Nonmetric Multi-dimensional Scaling (NMDS) plots of the pan-functional profiles of mouse fecal microbiome of the four experimental groups (white circle: WT mice on the VA-suf diet; gray circle: WT mice on the VA-def diet; light orange circle: *Lrat*^*−/−*^*Rbp*^*−/−*^ mice on the VA-suf diet; orange circle: *Lrat*^*−/−*^*Rbp*^*−/−*^ mice on the VA-def diet). B: Serum and tissue retinol and retinyl ester concentrations in *Lrat*^*−/−*^*Rbp*^*−/−*^ mice maintained for four weeks on the VA-def or VA-suf diet followed by the VA-suf diet for one week. Measurements by reversed-phase HPLC. Data are the mean ± SD; n = 3 mice/group. Statistical analysis by *t*-test. C: NMDS plots of the pan-functional profiles of mouse fecal microbiome of the *Lrat*^*−/−*^*Rbp*^*−/−*^ mice described in panel B (light orange circle: *Lrat*^*−/−*^*Rbp*^*−/−*^ mice before VA-suf; orange circle: *Lrat*^*−/−*^*Rbp*^*−/−*^ mice before the VA-def diet). NMD1 and 2, NMDS axes 1 and 2; VA-def, vitamin A deficient; VA-suf, vitamin A sufficient.

We next sought to predict the functional profile of the fecal microbiome of the *Lrat*^*−/−*^*Rbp*^*−/−*^ mice by annotating the 16S rRNA gene-based OTU of the fecal microbiome using QIIME (36) and mapping the detected taxa to fusionDB (39). The resulting set of data was used to generate a nonmetric multidimensional scaling plot to condense information from multidimensional data (many functions per microbiome) into a 2D representation where sample similarity is reflected in the plot distance between the corresponding points. Thus, the closer the points (i.e., samples) are together in the ordination space, the more similar the functionalities of the microbial communities are. Interestingly, here we observed a significant separation of microbial functionalities between WT (VA-suf and VA-def), *Lrat*^*−/−*^*Rbp*^*−/−*^ VA-suf, and *Lrat*^*−/−*^*Rbp*^*−/−*^ VA-def mice (PERMANOVA test, *P* < 0.001; Fig. 4A). In an effort to identify specific microbial functional differences between WT and mutant mice, we further extracted from the fusionDB functional profiles a list of experimentally verified bacterial functions (42) and mapped them to Kyoto Encyclopedia of Genes and Genomes molecular pathways. Notably, we found that enzymes involved in butanoate metabolism, known to be associated with VAD (22), were differentially represented in the *Lrat*^*−/−*^*Rbp*^*−/−*^ (VA-suf and Va-def) compared with the microbiome of WT VA-suf mice. Specifically, the genes differentially represented based on the Kyoto Encyclopedia of Genes and Genomes pathway database were acetolactate synthase I/II/III large subunit [EC:2.2.1.6], fumarate reductase flavoprotein subunit [EC:1.3.5.4], and formate C-acetyltransferase (*P* < 0.01, *t*-test). Thus, despite the similarity in fecal bacteria taxa between *Lrat*^*−/−*^*Rbp*^*−/−*^ mice on the VA-suf and VA-def diets, a clear phenotypic separation emerged between the two groups of mutant mice when microbial functionalities of the fecal samples were estimated. Next, two subsets of *Lrat*^*−/−*^*Rbp*^*−/−*^ mice maintained on the VA-suf or VA-def diets for four weeks (as above) were placed on a VA-suf diet for one week before collecting feces and tissues. Reintroducing the VA in the diet of the double-KO mice first restored serum and tissue retinoid levels of the mutants previously on the VA-def diet compared with those of the mutants continuously maintained on the VA-suf diet, that is, improved their VA status (Fig. 4B). Second, this regimen restored fecal microbial functionalities, that is, the two groups of *Lrat*^*−/−*^*Rbp*^*−/−*^ mice were no longer distinguishable based on their prior history of VA-def or VA-suf diets (PERMANOVA test, *P* < 0.146; Fig. 4C). These data suggested to us that the microbial functional segregation of the *Lrat*^*−/−*^*Rbp*^*−/−*^ mice on VA-suf and VA-def diets was driven by the more severe VAD of the mutant mice on the VA-def diet. Note that β-diversity was similar in these latter double-KO subgroups and those described in Fig. 3 (data not shown).

### Functional perturbations in the colon of the *Lrat*^*−/−*^*Rbp*^*−/−*^ mice

The fecal 16S rRNA gene survey of the *Lrat*^*−/−*^*Rbp*^*−/−*^ mice revealed a shift in abundance in bacteria taxa whose growth could be influenced, for instance, by mucus abundance, such as *Allobaculum*, *Prevotella*, *Disulfovibrio* (43), and *Mucispirillum* spp. (44, 45), by the levels of oxygen and/or ROS in the intestine, as in the case of *Prevotella*, *Paraprevotella*, and *Bacteroides* (46, 47) or by the inflammatory status of the host intestine, as for *Bacteroides* and *Helicobacter* spp. (48, 49). We therefore assessed whether functional perturbations, which could have created favorable niches for the above-mentioned bacteria to thrive, occurred in the mutant colon, where most of the bacteria reside.

**Fig. 5:**
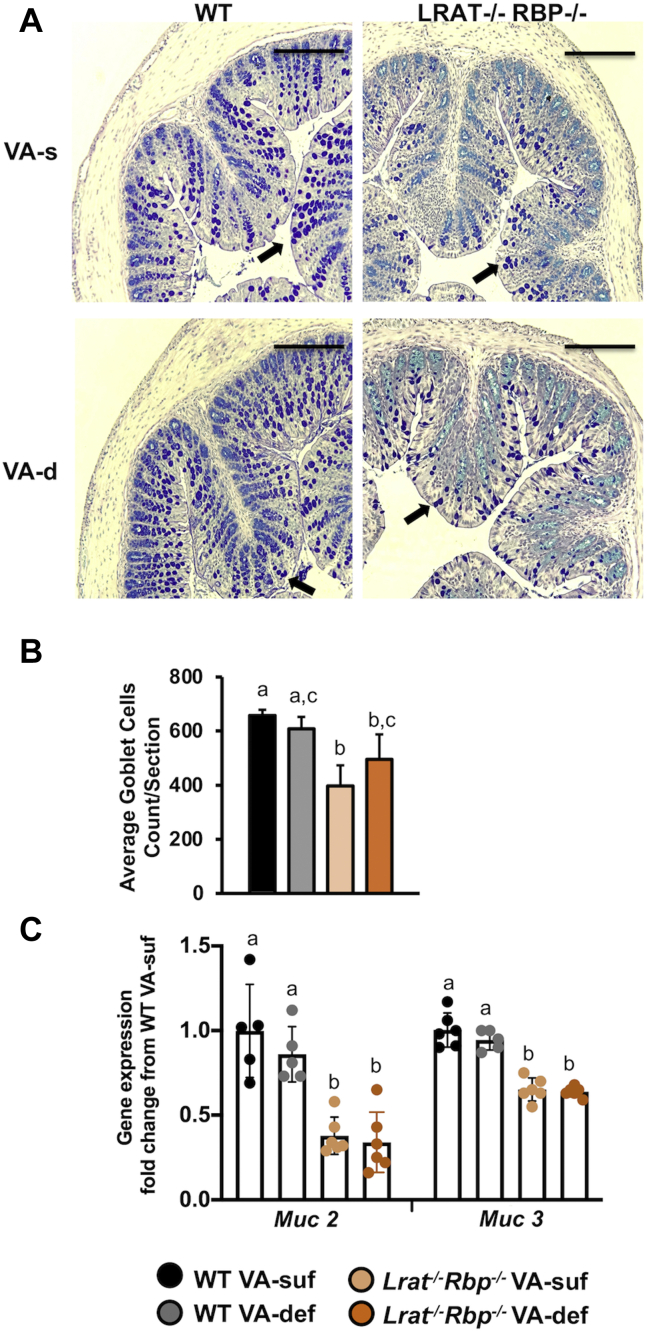
Goblet cells and mucins in the colon of *Lrat*^*−/−*^*Rbp*^*−/−*^ and WT mice. A: Alcian blue-periodic acids-Schiff (AB-PAS) staining for Goblet cells; scale bar = 220 μm; 100× magnification arrows point to goblet cells. Tissue embedding and sections as in Materials and Methods. B: Quantification of the number of goblet cells using three different depths with an average of 400 μm apart of six colon sections/mouse; n = 3 mice/group; data as mean ± SD. Different letters indicate significant differences among the groups (*P* < 0.05). Statistical analysis by two-way ANOVA C: Real-time RT-PCR analysis of mRNA expression levels of mucins (*Muc2* and *Muc3*). Data are the mean ± SD, calculated using the 2^−ΔΔ*CT*^ method; n = 5–6 mice/group. Statistical analysis by two-way ANOVA. Different letters indicate significant difference (*P* < 0.05) among groups. *Lrat*^−/−^, lecithin:retinol acyltransferase–deficient; *Rbp*^*−/−*^, retinol-binding protein–deficient; VA-def, vitamin A deficient; VA-suf, vitamin A sufficient.

The intestinal mucus layer, the first physical line of defense of the gastrointestinal tract, is mainly composed of mucins: *Muc* 2 (MUC2), produced selectively by the goblet cells, and *Muc* 3 (MUC3), expressed in both goblet cells and enterocytes (50). We found a significant reduction in the number of goblet cells, identified by PAS staining, in both *Lrat*^*−/−*^*Rbp*^*−/−*^ groups compared with the WT VA-suf group (Fig. 5A, B). Moreover, *Muc2* and *Muc3* expression was downregulated in the colon of *Lrat*^*−/−*^*Rbp*^*−/−*^ mice regardless of their dietary regimen compared with WT mice (Fig. 5C). Dietary VA deprivation of WT and *Lrat*^*−/−*^*Rbp*^*−/−*^ mice did not affect either the number of goblet cells (Fig. 5A, B) or the expression of the *Muc* genes (Fig. 5C).

**Fig. 6:**
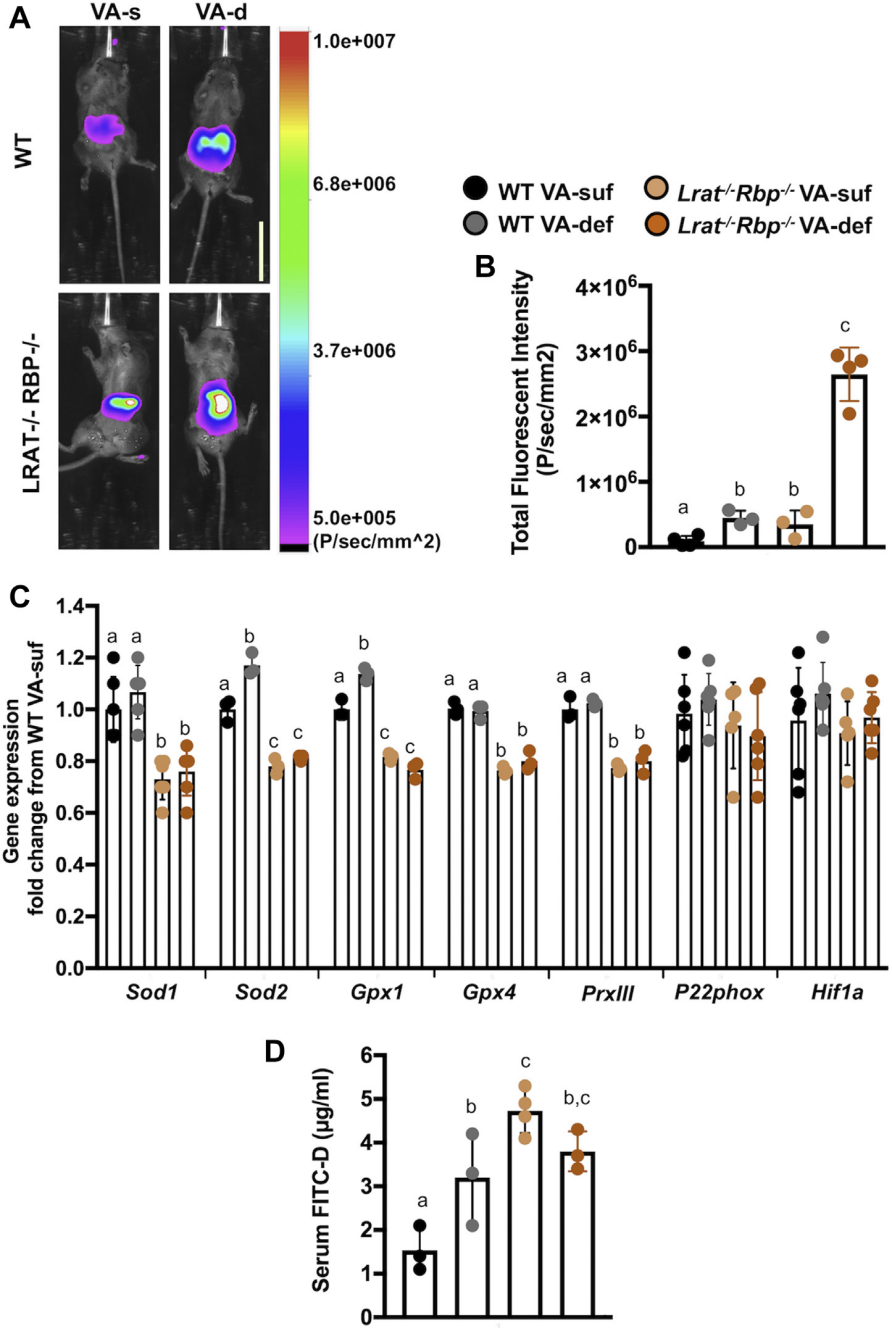
ROS and oxidative stress–related genes in the colon and serum FITC-dextran levels of *Lrat*^*−/−*^*Rbp*^*−/−*^ and WT mice. A: Overlay of ROS-associated near-infrared fluorescence (NIRF) image and corresponding brightfield image of WT (*top*) and *Lrat*^*−/−*^*Rbp*^*−/−*^ (bottom) mice. NIRF intensity scale is shown on the right. The image was normalized accordingly using Carestream Molecular Imaging software. White scale bar in the right bottom corner of the top right image = 3.6 cm. B: Quantification of the data in panel A. n = 4–5 mice/group. Values are the mean ± SD. Statistical analysis by two-way ANOVA. Different letters indicate significant difference (*P* < 0.05) among groups. C: mRNA expression levels of oxidative stress response genes (*Sod1*, *Sod2*, *Gpx1*, *Gpx2*, peroxiredoxins, cytochrome b-245 alpha chain, *Hif1a*) measured by real-time RT-PCR in the mouse colon. Values as the mean ± SD calculated using the 2^−ΔΔ*CT*^ method; 3 individual pools/group (six animals per pool). Statistical analysis by two-way ANOVA within each gene. Different letters indicate significant difference (*P* < 0.05) within each gene. D: Serum FITC-dextran levels in mice 4 h after gavage; n = 3 to 4 mice/group. Statistical analysis by two-way ANOVA. Different letters indicate significant difference (*P* < 0.05) among groups. ROS, reactive oxygen species; VA-def, vitamin A deficient; VA-suf, vitamin A sufficien.

Gut bacteria are sensitive to ROS and/or oxygen levels (46, 47). We therefore measured intestinal ROS levels by optical in vivo imaging using NIRF light generated by cyanine-based fluorescent dyes in WT and *Lrat*^*−/−*^*Rbp*^*−/−*^ mice. As indicated by the enhanced intensity of the fluorescent signal, ROS were significantly increased in the intestinal lumen of the *Lrat*^*−/−*^*Rbp*^*−/−*^ mice on both diets compared with WT mice on the VA-suf diet (Fig. 6A, B). However, *Lrat*^*−/−*^*Rbp*^*−/−*^ mice on the VA-def diet showed the highest fluorescent signal compared with all the other groups (Fig. 6A, B). Interestingly, lack of dietary VA also slightly but significantly increased luminal ROS in WT animals relative to the WT VA-suf group (Fig. 6B). To explore the contribution of the intestinal cells to the elevated luminal ROS of the mutant mice, we surveyed the expression of genes involved in oxidative stress regulation in the colon. The antioxidant genes *Sod1/2*, *Gpx1/4*, and peroxiredoxins were significantly reduced in the colon of the *Lrat*^*−/−*^*Rbp*^*−/−*^ mice compared with WT animals, regardless of the dietary VA content (Fig. 6C). Interestingly, dietary VA restriction resulted in increased expression of antioxidant genes, specifically *Sod2* and *Gpx1*, only in the WT (Fig. 6C). Overall, these data suggest that in the WT mice, by increasing ROS, dietary VA restriction triggers the antioxidant defense system in the colon. This system however seems impaired in the mutant mice likely contributing to the higher ROS levels in their intestinal lumen under the VA-def diet.

The increase in luminal ROS in the *Lrat*^*−/−*^*Rbp*^*−/−*^ mice also suggested an increased intestinal permeability in the mutant intestine, as well as in response to VA deprivation. To directly evaluate intestinal permeability, we used the FITC-dextran assay (32). As shown in Fig. 6D, serum FITC-dextran levels were significantly increased in *Lrat*^*−/−*^*Rbp*^*−/−*^ VA-suf and VA-def mice compared with WT VA-suf mice. Moreover, the dietary deprivation further enhanced these levels in the WT mice, but not in the mutants.

**Fig. 7:**
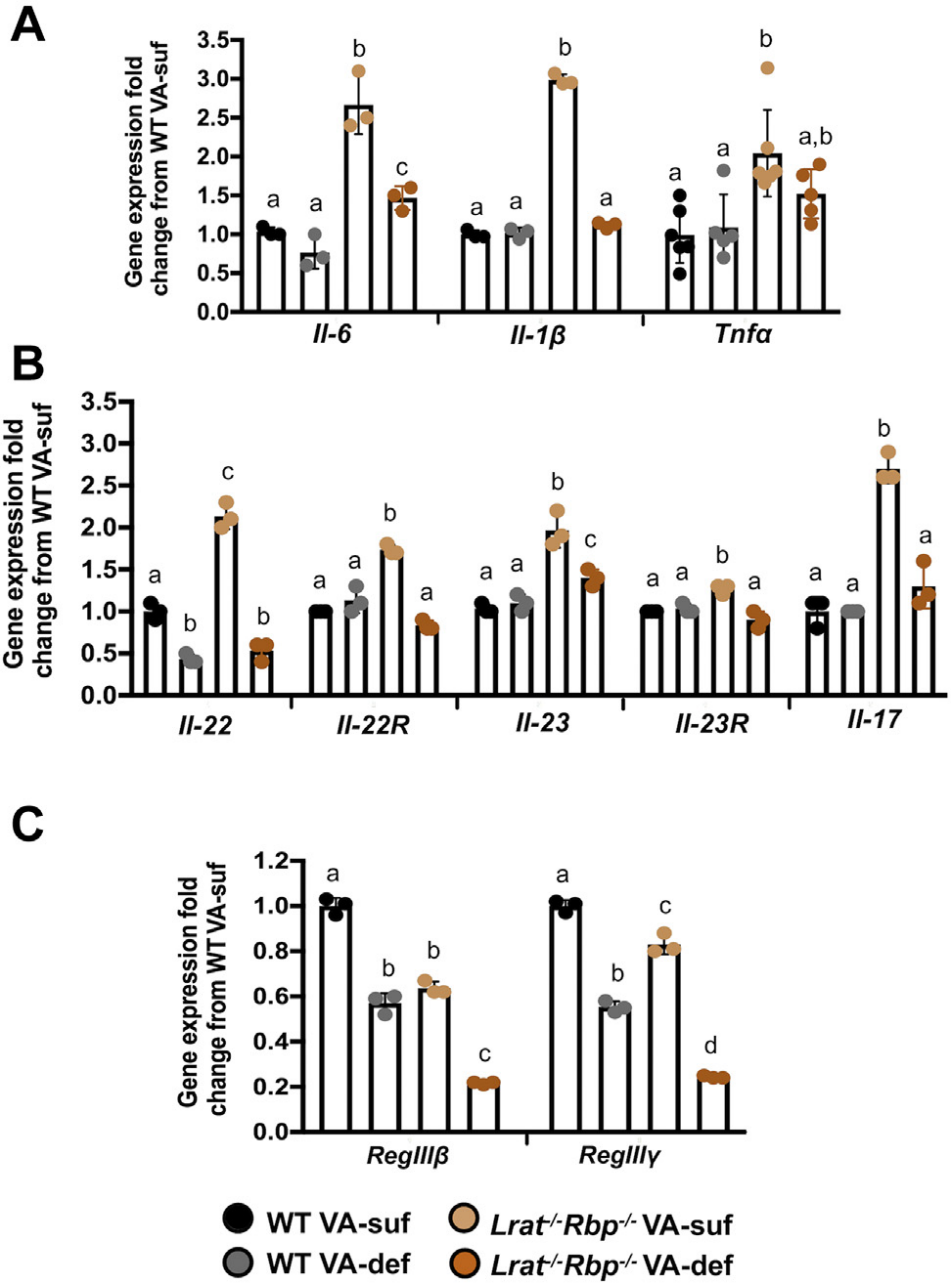
Inflammatory and immunomodulatory cytokines and antimicrobial proteins in the colon of *Lrat*^*−/−*^*Rbp*^*−/−*^ and WT mice. mRNA expression levels of (A) Inflammatory cytokines (*Tnfα*, *Il1β*, and *Il-6*); (B) immunomodulatory cytokines and their receptors (*Il22*, *Il22R*, *Il23*, *Il23R*, *Il13*, and *Il17*) and (C) antimicrobial proteins (*RegIIIβ* and *RegIIIγ*). Values are the mean ± SD calculated using the 2^−ΔΔ*CT*^ method; n = 5–6 mice/group or n = 3 individual sample pools/group (six animals per pool). Statistical analysis by two-way ANOVA within each gene. Different letters indicate significant difference (*P* < 0.05) among groups within each gene. *Lrat*^−/−^, lecithin:retinol acyltransferase–deficient; *Rbp*^*−/−*^, retinol-binding protein–deficient; VA-def, vitamin A-deficient diet; VA-suf, vitamin A-sufficient diet.

Inflammation has also been linked to intestinal dysbiosis (51). Message levels of key inflammatory markers, such as interleukin (*IL*)*-6* and tumor necrosis factor-alpha (*Tnfa*), were generally elevated in the colon of the *Lrat*^*−/−*^*Rbp*^*−/−*^ (VA-suf and VA-def) compared with both WT groups (Fig. 7A). Paradoxically, tumor necrosis factor (*Tnfa*)-alpha, *IL*-*1β*, and *IL-6* were significantly upregulated in the colon of the *Lrat*^*−/−*^*Rbp*^*−/−*^ mice on the VA-suf diet, not only compared with WT mice on both diets but also compared with *Lrat*^*−/−*^*Rbp*^*−/−*^ mice on the VA-def diet, suggesting a paradoxical increased inflammatory tone in the colon of the mutant mice fed the VA-containing diet. Gastrointestinal inflammation is often linked to aberrant expression of immunocytokines, especially *IL-22* and *IL-17* (52, 53). *IL-22* expression was significantly attenuated in the colon of WT and *Lrat*^*−/−*^*Rbp*^*−/−*^ mice on the VA-def diet (Fig. 7B), suggesting the dependence of this phenotype on the dietary VA intake. Surprisingly, this gene and the one encoding its receptor (II-22R) were significantly upregulated in the *Lrat*^*−/−*^*Rbp*^*−/−*^ VA-suf group (Fig. 7B). Moreover, expression levels of *IL-23*—an inducer of *IL*-22 (54)—increased in the *Lrat*^*−/−*^*Rbp*^*−/−*^ mice on both diets, whereas a slight but significant upregulation of its receptor (*IL-23R*) could be observed in the *Lrat*^*−/−*^*Rbp*^*−/−*^ VA-suf group (Fig. 7B). *II-17* mRNA levels were also significantly increased exclusively in *Lrat*^*−/−*^*Rbp*^*−/−*^ mice on the VA-suf diet compared with all other groups (Fig. 7B). Overall, the aberrant expression of *IL-22* and *IL-17* in the colon of *Lrat*^*−/−*^*Rbp*^*−/−*^ mice on the VA-suf diet agrees with the paradoxical increased inflammatory tone we observed in their colon.

IL-22 is also a key enhancer of the expression of antimicrobial proteins, such as regenerating islet-derived protein 3 (*RegIII*)*β* and *RegIIIγ*. These proteins are synthesized throughout the intestine by different cell types for control of the number of luminal bacteria (55). In agreement with the lower expression of *IL-22* (Fig. 7B), the expression of both *RegIIIβ* and *RegIIIγ* genes was significantly attenuated in the colon of the *Lrat*^*−/−*^*Rbp*^*−/−*^ mice compared with WT mice on the VA-suf diet (Fig. 7C). VA deprivation significantly reduced *RegIIIβ* and *RegIIIγ* mRNA levels in both genotype (Fig. 7C) and *Lrat*^*−/−*^*Rbp*^*−/−*^ mice on the VA-def diet showed the lowest expression levels of these two genes compared with all other groups (Fig. 7C).

Overall, these data show functional differences between the WT and mutant colon, some of which were heightened by the lack of VA in the *Lrat*^*−/−*^*Rbp*^*−/−*^ mice. These results indicate that the dietary VA restriction results in structural damage in the intestinal epithelium of the WT mice as well.

## Discussion

The goal of this study was to gain insights into the impact of impaired VA transport and stores formation on intestinal retinoid homeostasis, fecal microbiome diversity, as well as intestinal barrier functions by using mice lacking both *Lrat* and *Rbp*. Our data confirmed that the precarious VA status of this mutant strain is further compromised under a restricted dietary VA intake (11, 25), and showed that this is also the case in peripheral tissues such as the intestine. We found that LRAT is the major enzyme that esterifies ROH in the duodenum, in agreement with O'Byrne *et al.* (10). The residual REs observed in the mutant's small intestine are likely due to the activity of DGAT1 (40). Despite similar expression levels in the colon and intestine (*Lrat* quantitative real-time PCR CT values were 28.3 ± 0.3 and 27.5 ± 1.2 in WT colon and duodenum, respectively; n = 6/group), LRAT does not seem to be the predominant ROH-esterifying enzyme in the colon, where RE levels are in fact comparable between mutant and WT mice on the VA-containing diet (Fig. 1). It is likely that DGAT1 also functions to esterify ROH in the colon, but it remains to be unequivocally proved. As most of the VA absorption occurs in the small intestine (7), it was not surprising that RE levels dropped significantly in the WT duodenum upon dietary VA restriction (Fig. 1). Interestingly, in the WT strain, the magnitude of the RE reduction induced by the lack of dietary VA was greater in the small intestine (88%) than in the colon (35%) (compare Fig. 1H and Fig. 1K). These data confirm a less pronounced contribution of the newly absorbed dietary VA to the total ROH content of the colon compared with the duodenum, reflecting the limited role of the colon in the absorption of dietary VA. STRA6, the receptor for the ROH-RBP complex (56, 57), is expressed in the small intestine (24), as well as in the colon, as suggested by a human colonic cell line (58). Thus, it is reasonable to hypothesize that the VA mobilized from the hepatic stores as ROH-RBP is delivered to both segments of the gut, hence the presence of ROH and REs—although at reduced levels—in the duodenum and colon of WT mice deprived of dietary VA (Fig. 1). In the mutant mice, that is, in the absence of RBP, retinoids could still be delivered to the gastrointestinal tract by RE-containing chylomicrons, as 25% of these postprandial lipoprotein particles are cleared from the circulation by extrahepatic tissues (7). Indeed, ROH and REs become undetectable in the intestine of the mutant mice on the VA-def diet (Fig. 1). Clearly, dietary VA maintains RA homeostasis in the tissues of the mutant mice, including the intestine, despite their extremely low levels of serum ROH and tissue retinoid stores. The dietary VA restriction however attenuated the concentration of RA in the tissues of the *Lrat*^*−/−*^*Rbp*^*−/−*^ mice, including the duodenum and colon, exacerbating their VA deficient status. Note that even smaller fluctuations in the concentration of a transcriptional regulator, such as RA, can have a large impact on overall cell and tissue functions.

It is becoming evident that VAD compromises the intestinal microbiome (21, 22, 23), although the distinction between the primary effects of the dietary micronutrient content versus the host health-related effects has remained still difficult to distinguish. By using a gnotobiotic mouse model of human gut microbiome development, Hibberd *et al*. (23) proposed that micronutrient deficiencies (such as few weeks of dietary VA deprivation) disrupt microbiome development directly. However, in the Hibberd study (23) the VA status of the mice was not rigorously assessed, making the interpretation of the effects of the dietary manipulation challenging, especially in a model with a compromised gastrointestinal tract, such as the gnotobiotic mice (59). Recently, Tian *et al*. (22) induced VAD in WT mice by maintaining the mothers on the VA-def diet during pregnancy and the weanlings on the same diet up to 12 weeks of age. In agreement with other reports in mice and humans (21, 23), and based on cecal 16S rRNA gene sequencing, these authors showed that fewer *Bacteroidetes* and fewer butyrate-producing bacteria were present in the intestine of the VAD mice and proposed that “changes in microbiome are caused by VAD” (22). Our findings support this relationship. It is possible that the differences in the taxonomic footprint of the fecal bacteria community observed between mutant and WT mice on the VA-suf diet could have arisen form yet undiscovered local actions of RBP and/or LRAT in the intestine. However, the fecal microbiome taxonomic differences in the mutant mice were similar to those observed in VA deficient humans and mice. For instance, the mutant mice showed lower fecal abundance of bacteria with known antioxidant and anti-inflammatory properties, including *Bifidobacterium* sp., and a greater abundance of bacteria with known proinflammatory impact, including *Bacteroides* sp. (Table 1), as well as reduced fecal SCFA (Fig. 3) (21, 22, 23, 60). Therefore, we argue that the systemic differences in VA storage, metabolism, and mobilization could have played a major role in shaping the fecal microbiome of the double-KO mice, reflecting the precarious VA status of the mutant mice. Indeed, WT mice did not develop VAD when maintained on the VA-def diet for four weeks (Fig. 1), and both the PCA and the functional analysis of the 16S rRNA gene sequences clearly distinguished them from the mutant mice. Moreover, the two WT groups could not be separated based on the VA concentration in the diet (Figs. 2 and 4). These findings indicate that the amount of VA in the diet per se does not directly influence fecal bacterial diversity. This is reasonable if one considers that absorption of dietary VA is not a regulated process; in other words, the more VA is ingested, the more is absorbed (7). Thus, the amount of unabsorbed VA that travels from the small intestine through the colon to be ultimately excreted with the feces is normally negligible (9). Importantly, the analysis of the fecal microbial functionalities with fusionDB (39) not only confirmed the distinction of the mice based on their genotype, that is, the VA status (sufficient vs. deficient) but also clearly distinguished the two groups of *Lrat*^*−/−*^*Rbp*^*−/−*^ mice (Fig. 4). This latter separation was not due to the VA content of the diet per se, as it was not seen in the WT mice. Therefore, we inferred that it should be linked to the exacerbated VAD of the mutant mice secondary to the dietary VA restriction (Fig. 1). Indeed, replenishing the VA in the diet of the VA-def *Lrat*^*−/−*^*Rbp*^*−/−*^ group for one week restored serum and tissue retinoid concentrations to their baseline levels and abolished the separation of microbial functionalities observed between VA-def and VA-suf *Lrat*^*−/−*^*Rbp*^*−/−*^ mice (Fig. 4). These findings further support our interpretation that the VA-def status of the host (mutants) impacted the fecal microbiome.

This work was conducted with female mice. Thus, given the influence of sex hormones and estrous cycle on the microbiome (61), it still needs to be understood whether these effects of the VA-def host may be sex dependent. In the future, these studies will also need to be extended to the microbiome of the intestine, including its mucosa, which is emerging as a critical factor in defining bacteria–host interactions (62). Ideally, these studies should also be conducted with co-housed F2 littermates to minimize variations in microbiota that could confound genotype–phenotype studies.

Although H&E staining did not reveal gross morphological defects in the colon of the *Lrat*^*−/−*^*Rbp*^*−/−*^ mice (data not shown), the functionality of the gut barrier was compromised in the mutant mice, already when they were fed the VA-suf diet. These dysfunctions could have potentially created favorable niches for certain bacteria to thrive under higher levels of ROS and oxygen and/or intestinal inflammation and/or reduced mucus (Table 1). Although we cannot rule out that these functional phenotypes of the mutant colon might have arisen from VAD during embryogenesis, given the precarious VA status of this strain (11, 25), some of them clearly distinguished the two mutant groups secondary to the dietary VA restriction that resulted in a more severe VAD of the *Lrat*^*−/−*^*Rbp*^*−/−*^ mice.

Barrier function depends primarily on the mucus layer that separates the epithelial cells from the luminal content, thereby providing a niche for the intestinal bacteria to colonize the gut (50). Previous studies have shown that *Muc* expression (*Muc2* and *Muc5*) is regulated by RA, at least in human epithelial cells in vitro (63, 64). In the small intestine of VAD mice, *Muc2* mRNA levels increased (65). In contrast, in the colon of VAD rats, *Muc2* expression was reduced by 75%, whereas *Muc3* was increased (66), suggesting region- and species-specific differences within the gastrointestinal tract. Given that colon RA concentrations were not significantly different between WT and *Lrat*^*−/−*^*Rbp*^*−/−*^ mice on the VA-suf diet (Fig. 1), it seems unlikely that the reduced expression of the *Muc* genes in the mutant mice arose from a RA–dependent transcriptional repression of *Muc2/3*, at least when the mice were reared on the VA-containing diet. Perhaps, it could be due to the reduced number of goblet cells in the mutants (Fig. 5), for example, as a legacy of VAD during embryogenesis (11, 25). Nevertheless, this reduction in *Muc* could have altered the intestinal mucus layer (67) inhibiting the growth of *Muc*-dependent bacteria, such as *Allobaculum*, while favoring the abundance of bacteria less dependent on *Muc* to thrive, such as *Prevotella* (Table 1) (43).

VAD has been associated with oxidative stress (68, 69, 70), which in turn results in increased generation of ROS that, together with oxygen, could diffuse into the gut lumen from a structurally compromised intestinal epithelium, disturbing the bacterial populations in the gut (33). Higher levels of ROS in the intestinal lumen (Fig. 6C) and imbalance in the mucosal expression of key oxidative stress genes (Fig. 6B) suggest increased oxidative stress in the colon of the mutant mice already on the VA-suf diet. Although increased levels of ROS are normally associated with higher expression of antioxidant genes, a number of studies have also shown that oxidative stress may increase as a result of impaired antioxidant activity of enzymes such as superoxide dismutase and glutathione peroxidase (71, 72, 73). Thus, the lower expression of the antioxidant-related genes in the colon of the VAD mutants (Fig. 6C) does not contradict our interpretation of an increased oxidative stress in the *Lrat*^*−/−*^*Rbp*^*−/−*^colon. Likely, the ROS “leaked” from the compromised intestinal barrier (Fig. 6D). The dietary VA deprivation of the *Lrat*^*−/−*^*Rbp*^*−/−*^ mice clearly exacerbated the rise in intestinal ROS (Fig. 6C), possibly as a result of the inability of the mutant intestinal cells to activate a proper antioxidant defense, as seen in the WT (Fig. 6C).

*RegIIIβ* and *RegIIIγ* are two antimicrobial proteins that control bacterial load in the gut (55). Their expression was compromised in the colon of the mutant mice, even more so when their VA-def status was exacerbated by the dietary VA deprivation (Fig. 7). It is unknown whether these genes are directly regulated by retinoids. However, it is established that the mucosal antimicrobial response of the intestinal epithelial cells is modulated by IL-22, an immunomodulatory cytokine synthesized by various cells of the immune systems, including innate lymphoid cells, T lymphocytes, and dendritic cells (53). Interestingly, RA promotes maturation and proliferation of IL-22–producing RORγt + innate lymphoid cells, directly binds to the II-22 promoter, and upregulates IL-23 from dendritic cells that acts as a potent inducer of IL-22 (as reviewed (24)). Thus, the lower expression of *II-22* in the colon of the mutants on the VA-def diet is in agreement with the compromised antimicrobial response of these mice (Fig. 7) and is likely linked to their attenuated RA concentrations in the colon mucosa (Fig. 1). Surprisingly, however, the colon of the *Lrat*^*−/−*^*Rbp*^*−/−*^ mice on the VA-suf diet showed enhanced expression of *II-22* and its inducer, *IL-23*, as well as *II-17*, all potential drivers of chronic gastrointestinal inflammation (52, 53). Indeed, the colon of the *Lrat*^*−/−*^*Rbp*^*−/−*^ mice on the VA-suf diet also presented enhanced expression of inflammatory cytokines (Fig. 7). Although IL-22 has been shown to positively regulate intestinal barrier functions (74), its induction is not always beneficial for the host, as it could favor infections by pathogens such as *Salmonella* (75), or lead to pathological inflammation (76, 77). Interestingly, it has been shown that under a condition of altered microbial load, RA can promote, rather than prevent, the inflammatory response (78). We therefore hypothesize that the paradoxical higher inflammatory tone of the colon of the *Lrat*^*−/−*^*Rbp*^*−/−*^ mice on the VA-suf diet could be sustained by a synergistic effect of the relatively normal levels of RA and the potentially altered microbial-driven signal(s) in the gastrointestinal tract of mutant mice on the VA-suf diet. Note that the inflammatory status of other organs from these mice was not investigated, and we cannot exclude a more widespread inflammatory condition.

In conclusion, our work showed that the intestine of mice lacking both *Lrat* and *Rbp* is susceptible to VAD, which is accompanied by fecal dysbiosis and functional perturbations of the barrier in the colon. We also demonstrated that the dietary VA deprivation, which heightens the VA insufficiency of the mutant mice—systemically and locally (in the liver and gastrointestinal tract)—further distinguished the two group of *Lrat*^*−/−*^*Rbp*^*−/−*^ mice based on fecal microbial functionalities as well as ROS levels, inflammatory status, and antimicrobial defense mechanisms in the colon. Surprisingly, whereas the dietary VA deprivation did not result in VAD or fecal dysbiosis or inflammation in the WT mice, it did attenuate RA concentration in the colon and modulated key components of the intestinal barrier, implying the development of functional changes in this segment of the intestine. Indeed, the VA-restricted WT mice showed signs of compromised gut integrity as indicated by the modest but significant increase in luminal ROS (Fig. 6) and serum levels of FITC-dextran (Fig. 6), as well as by the reduced mRNA expression of the antimicrobial proteins and the immunomodulatory cytokine IL-22 (Fig. 7). These phenotypes might be linked to the reduction in the RA concentration that occurred in the colon of the VA-restricted WT mice, despite their systemic VA sufficiency (Fig. 1). Whereas it is commonly believed that tissue RA deficiency can only be achieved in the context of overt VAD, some reports suggest that this may not be the case. For instance, attenuation of RA signaling has been reported in the lung of WT mice fed a VA-def diet only for 5 days, in the absence of systemic VAD (79). Our data are in agreement with this emerging notion, although the true physiological significance of these diet-induced changes in the context of the biology of the colon and the basis for this phenomenon remain to be addressed. Overall, our findings reveal the suitability of *Lrat*^*−/−*^*Rbp*^*−/−*^ mice as a model to study intestinal dysfunctions and dysbiosis depending upon changes in tissue retinoid homeostasis induced by the host VA intake and status.

### Data availability

The fecal 16S rRNA gene survey raw data have been deposited in the NCBI's Sequence Read Archive (SRA) data repository (BioProject number PRJNA679057) and can be downloaded without any restrictions. All the remaining data are contained within the article.

## Supplemental data

This article contains supplemental data.

## Conflict of interest

The authors declare that they have no conflicts of interest with the contents of this article.
