# Supplementary material for: Impact of vitamin A transport and storage on intestinal retinoid homeostasis and functions
Source: J Lipid Res. 2021 Feb 13;62:100046. doi: 10.1016/j.jlr.2021.100046 (PMC8020483; doi:10.1016/j.jlr.2021.100046)
Supplement: Supplemental Tables S1 & S2 and Figures S1 & S2 [file mmc1.pdf]

SUPPLEMENTAL INFORMATION

Impact of vitamin A transport and storage on intestinal retinoid homeostasis and functions

Maryam Honarbakhsh[1], Aaron Ericsson[2], Guo Zhong[3], Nina Isoherranen[3], Chengsheng Zhu[4], Yana Bromberg[4], Charlene Van Buiten[5], Kiana Malta[1], Laurie Joseph[6], Harini Sampath[7,8], Atreju I. Lackey[7], Judith Storch[7,8], Costantino Vetriani[4], Michael L. Chikindas[1], Paul Breslin[7], Loredana Quadro[1, 8, *]

[1]Department of Food Science, Rutgers University, New Brunswick, NJ, USA
[2]Department of Veterinary Pathobiology, University of Missouri Metagenomics Center, University of Missouri, Columbia, MO, USA
[3] Department of Pharmaceutics Health Sciences, University of Washington, Seattle, WA, USA
[4]Department of Biochemistry and Microbiology, Rutgers University, New Brunswick, NJ, USA
[5]Department of Plant Biology, Rutgers University, New Brunswick, NJ, USA
[6]Department of Pharmacology and Toxicology, Rutgers University, Piscataway, NJ, USA
[7]Department of Nutritional Sciences, Rutgers University, New Brunswick, NJ, USA
[8]Rutgers Center for Lipid Research and Institute of Food Nutrition and Health, Rutgers University, New Brunswick, NJ, USA

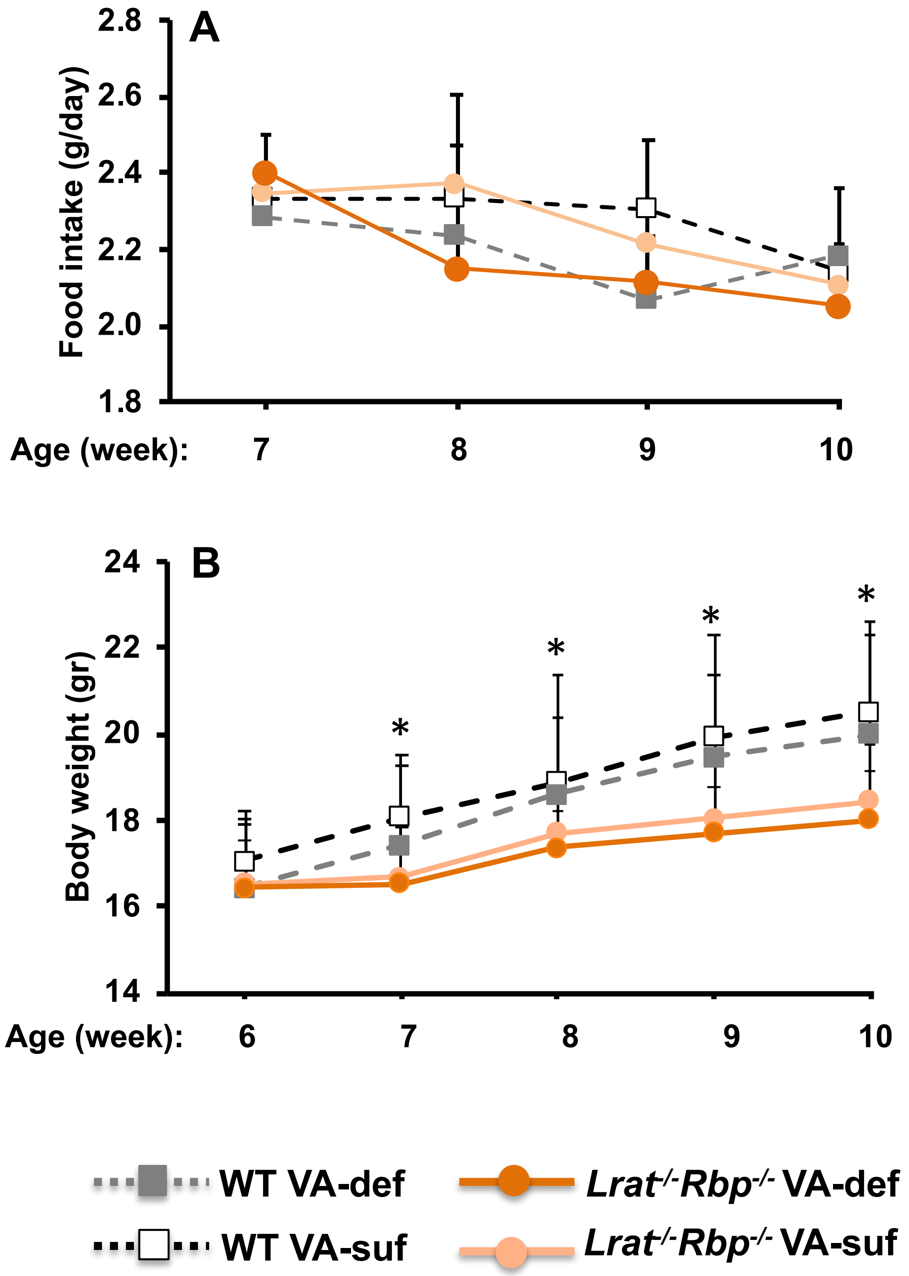

Supplemental Fig. S1. Food intake and body weight change in *Lrat*$^{-/-}$*Rbp*$^{-/-}$ and WT mice throughout the experiment. (**A**) Food intake and (**B**) body weight throughout the experiment. Week indicates the age of the mice. Values are mean ± SD. Statistical analysis by two-way ANOVA at each time point; *, $p < 0.05$ indicates significant differences between *Lrat*$^{-/-}$*Rbp*$^{-/-}$ (VA-suf and VA-def) *vs.* the reference group WT VA-suf. VA-def, vitamin A-deficient diet; VA-suf, vitamin A-sufficient diet.

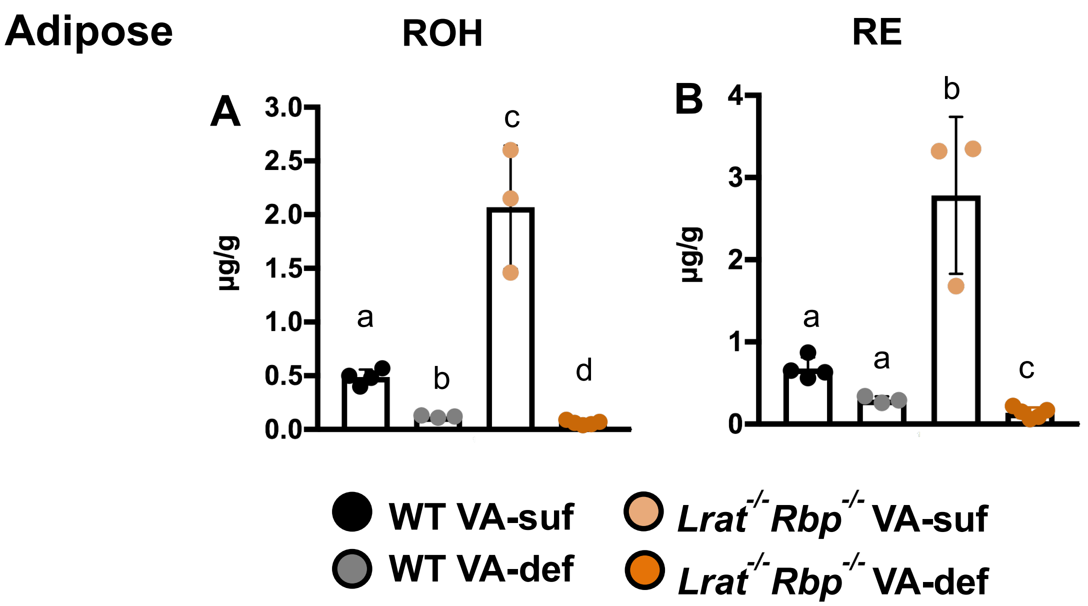

Supplemental Fig. S2. Retinol and retinyl ester levels in adipose tissue in *Lrat*$^{-/-}$*Rbp*$^{-/-}$ and WT mice throughout the experiment. (**A**) retinol (ROH) and (**B**) retinyl ester (RE) levels were determined by reversed-phase HPLC analysis. Data are mean ± SD; n = 3 - 5 mice/group. Statistical analysis by two-way ANOVA for normally distributed data and by Mann–Whitney test for non-normally distributed data. Statistical analysis by two-way ANOVA for normally distributed data and by Mann–Whitney test for non-normally distributed data. Different letters indicate significant differences ($p < 0.05$) among the groups. VA-def, vitamin A-deficient diet; VA-suf, vitamin A-sufficient diet.

Table S1. Primer sequences used for real-time PCR analysis			
**Gene**	**Forward Primer (5'-3')**	**Reverse Primer (5'-3')**	**Reference**
Tbp	CAAACCCAGAATTGTTCTCCTT	ATGTGGTCTTCCTGAATCCCT	Harvard Primer Data Bank
Muc2	GTCTGCCACCTCATCATGGA	CAGGCAAGCTTCATAGTAGTGCTT	This Study
Muc3	GTGGGACGGGCTCAAATG	CTCTACGCTCTCCACCAGTTCCT	This Study
RegIIIβ	GGCTTATGGCTCCTACTGCTATG	ATGGAGGACAAGAATGAAGCC	PMID: 27183576
RegIIIγ	GCTCCCGTGCCTATGGCTC	ATCATGGAGGACAGGAAGGAAG	PMID: 27183576
Il- β	CAACCAACAAGTGATATTCTCCATG	GTGCCGTCTTTCATTACACAG	Harvard Primer Data Bank
Il-6	TCGGAGGCTTAATTACACATGTTC	TGCCATTGCACAACTCTTTTCT	This Study
Tnf-a	TCGGAGGCTTAATTACACATGTTC	TGCCATTGCACAACTCTTTTCT	Harvard Primer Data Bank
Il-22	CGAGGAGTCAGTGCTAAGGATCAGTG	GATTGCTGAGTTTGGTCAGGAAAGG	PMID: 27183576
Il-22(R)	AGCTCGTGTATCTCTGACGC	TTGGCTCTGTCCATACATCTTGT	This Study
Il-23	AATAATGTGCCCCGTATCCAGT	GCTCCCCTTTGAAGATGTCAG	This Study
Il-23(R)	TGCATGTGGTGATAGCCCTTT	AGGCTCAACCCACATGTCAC	This Study
Il-17	CCCATGGGATTACAACATCACTC	CACTGGGCCTCAGCGATC	PMID: 20434372
Il-13	CACACAAGACCAGACTCCCC	GTTGGTCAGGGAATCCAGGG	This Study
Harvard Primer Data Bank: http://pga.mgh.harvard.edu/primerbank/			

**Table S2. Alpha diversity by Shannon Index, Simpson and Chao-1**				
	WT (VA-suf)	WT (VA-def)	*Lrat*$^{-/-}$*Rbp*$^{-/-}$(VA-suf)	*Lrat*$^{-/-}$*Rbp*$^{-/-}$(VA-def)
**Shannon Index**	3.9 ± 0.1	3.7 ± 0.2	4.1 ± 0.1	3.9 ± 0.2
**Simpson**	0.96 ± 0.01	0.95 ± 0.01	0.96 ± 0.01	0.96 ± 0.02
**Chao-1**	367.44 ± 52.1	329.26 ± 39	348.32 ± 58.1	342.94 ± 61.1
Values are mean ± SD, n=5 mice per group				
